# Indeterminate Form of Chagas Disease: Some Immunological Insights

**DOI:** 10.1590/0037-8682-0594-2021

**Published:** 2022-04-29

**Authors:** Daniel Mazza Matos, Carlos Eduardo Menezes Viana, Maria de Fátima Oliveira, José Ajax Nogueira Queiroz

**Affiliations:** 1 Universidade Federal do Ceará, Departamento de Patologia e Medicina Legal, Fortaleza, CE, Brasil.; 2 Universidade Federal do Ceará, Departamento de Análises Clínicas e Toxicológicas, Fortaleza, CE, Brasil.

**Dear Editor:**

We read with interest the article by Hasslocher-Moreno and colleagues about the indeterminate form of Chagas Disease (CD)^1^.

As noted by the authors, the major characteristic of the indeterminate form of CD is its latent period. Approximately half of the patients remain indefinitely in the indeterminate form of the disease. More importantly, from a pathophysiological point of view, it remains unclear why some patients, after the acute phase of CD, manifest cardiac or gastrointestinal symptoms (or even a combination of them), while the majority remain in the indeterminate form, with a life expectancy similar to that of uninfected participants^2^ ^,^ ^3^. Factors related to *Trypanosoma cruzi* infection in areas with sustained vector transmission, male sex, parasite load, genetic aspects of the host, nutritional status, comorbidities, and the social context and quality of life of patients with CD are involved^1^. In addition, we believe that an imbalance between some cell actors in the host’s immune system and the parasite is also involved. 

Monocytes/macrophages and neutrophils are the first lines of defense against pathogens^4^. In the context of CD, specialized literature has emphasized the role of macrophages in controlling parasite growth. In an opposite line of investigation, there has been shown that some cytokines, for example, interleukin-4 (IL-4), prevent unwanted excessive tissue inflammation^5^.

Recently, we investigated the role of a specific subtype of monocyte/macrophage lineage cells, known as immunosuppressive CD14^+^/HLA-DR^low/‒^ monocytes, in 57 patients with 3 different clinical forms of chronic CD: 26 cardiac, 23 indeterminate, and 8 mixed patients^6^. These monocytes are a kind of myeloid-derived suppressor cells able to suppress the response of T-lymphocytes using the process of direct cell-cell interactions and the production of cytokines^7^. We have shown that patients with the indeterminate form can be distinguished by the predominance of CD14^+^/HLA-DR^low/‒^ monocytes compared with patients with cardiac and mixed forms. 

Given the immunosuppressive nature of these monocytes, our findings provide evidence that this specific population of cells is involved in the immunological response to the indeterminate form of CD, possibly influencing the control of severe inflammation carried out by *T. cruzi* in the host tissues. Significantly, it is possible to suggest that the predominance of CD14^+^/HLA-DR^low/-^ monocytes might have an immunoregulatory role inside the immune system that could ultimately delay (or even prevent) the evolution of the indeterminate form to the symptomatic forms (cardiac and mixed) of CD^6^.

Another important part of the immunological response against *T. cruzi* is represented by the cellular arm of the host’s immune system, namely, CD4^+^ and CD8^+^ T-lymphocytes and extrathymic double-positive CD4^+^/CD8^+^ T-lymphocytes^8^ ^,^ ^9^. More specifically, CD8^+^ T-lymphocytes are probably the most important effector cells in parasite control^10^. 

Therefore, taking advantage of our cases of CD, we studied the frequency of T-lymphocyte subpopulations in 57 patients: 34 with symptomatic clinical forms (26 cardiac and 8 mixed) and 23 with indeterminate form. All patients had previously received treatment with benznidazole (5 mg/kg/day) between 2011 and 2016. This study was approved by the Research Ethics Committee of the Federal University of Ceará.

We studied the T-lymphocyte subpopulations using flow cytometry. Briefly, approximately 4 mL of blood was collected using ethylenediaminetetraacetic acid (EDTA). In the incubation procedure, 1.0 × 10^6^ leukocytes were pipetted into one tube along with 5 μL of anti-CD3 (FITC), anti-CD8 (PE), and anti-CD4 (PerCP) monoclonal antibodies. The tube was incubated for 20 min at room temperature and protected from light. Next, the erythrocytes were lysed by adding 2 mL of 1× ammonium chloride lysing solution. Then, 100 μL of phosphate-buffered solution (PBS) was added, and the tube was washed twice by centrifugation at 1,500 rpm for 5 min. The supernatant was discarded, and the pellet was resuspended in 500 μL of PBS. The acquisition was carried out using a BD FACSCalibur™ flow cytometer.

Quantification of CD4^+^, CD8^+^, and CD4^+^/CD8^+^ T-lymphocytes was carried out through the analysis of dot plots showing the CD3, CD4, and CD8 antigens. In addition, the total lymphocyte count (μL) was determined using ADVIA 2120i hematology equipment.

One-way analysis of variance (ANOVA) and Tukey’s multiple comparisons were used to compare the different T-lymphocyte subpopulations. Differences were considered statistically significant at *P* < 0.05. All tests were performed using GraphPad Prism, version 6.0 (GraphPad Software, San Diego, USA).

Table 1 shows the results for the T-lymphocyte subpopulations. There was no statistical difference between the cardiac, mixed, and indeterminate forms of CD.

**TABLE 1:** CD4^+^ and CD8^+^ T-lymphocytes subpopulations in the different clinical forms of CD.

	Forms of Chagas Disease*						
T-lymphocytes subpopulations	Cardiac		Indeterminate		Mixed		*P*
	M	DP	M	DP	M	DP	
Total lymphocytes	1,634	425.5	1,633	529.7	1,628	434.9	0.9994
T-CD4^+^	830.3	573.3	713.4	240.9	691	233.7	0.5577
T-CD8^+^	420.2	153.5	379.2	184.6	365	184.1	0.614
CD4/CD8 ratio	1.84	0.52	2.11	0.79	2.31	1.07	0.2083
T-CD4^+^/CD8^+^	67.94	134.5	26.19	16.78	33.9	21.64	0.4443

**M:** mean; **SD:** standard deviation. *Values are ‘cells/μL’.

The central role of CD8^+^ T-lymphocytes in CD is related to the cytotoxic capacity of these cells, making them essential for infection control^10^. Unfortunately, we did not find any quantitative differences between the three groups. Our data confirm the previous study of Dutra et al., which did not find differences between the percentages of CD4^+^ and CD8^+^ T-lymphocytes between patients with cardiac and indeterminate forms of CD^8^.

A possible explanation for Dutra and our findings could be related to the tropism of some populations of CD8^+^ T-lymphocytes to the cardiac tissue^11^. It has been previously shown that CD8^+^ T-lymphocytes predominate over CD4^+^ T-lymphocytes in the cardiac tissue of patients with chronic CD^12^. Therefore, it is possible to speculate that the absence of quantitative differences between the subpopulations of CD4^+^ and CD8^+^ T-lymphocytes in the blood of patients with chronic CD does not reflect the intricate relationship between these cells (and other cellular subtypes of the host’s immune system) and *T. cruzi* at the sites of organ injury.

Chagas disease immunology is a jigsaw puzzle whose pieces have recently begun to fall into place^4^. For future studies, a particularly interesting project could focus on the possible relationship between immunosuppressive monocytes and the cellular arm of the host’s immune system. If the participation of some cell subtypes and cytokines with immunosuppressive functions and properties, respectively, can be observed in patients with CD, it is possible to understand why some patients evolve to symptomatic forms, while a subgroup of patients with the indeterminate form remains indefinitely asymptomatic.

## References

[1] Hasslocher-Moreno AM, Xavier SS, Saraiva RM, Sousa AS (2021). Indeterminate form of Chagas disease: historical, conceptual,
clinical, and prognostic aspects. Rev Soc Bras Med Trop.

[2] Coura JR, Borges-Pereira J (2012). Chagas disease. What is known and what should be improved: a
systematic review. Rev Soc Bras Med Trop.

[3] Nunes MC, Dones W, Morillo CA, Encina JJ, Ribeiro AL (2013). Council on Chagas Disease of the Interamerican Society of
Cardiology. Chagas disease: an overview of clinical and epidemiological
aspects. J Am Coll Cardiol.

[4] Acevedo GR, Girard MC, Gómez KA (2018). The Unsolved Jigsaw Puzzle of the Immune Response in Chagas
Disease. Front Immunol.

[5] Soares MB, Silva-Mota KN, Lima RS, Bellintani MC, Pontes-de-Carvalho L, Ribeiro-dos-Santos R (2001). Modulation of chagasic cardiomyopathy by interleukin-4:
dissociation between inflammation and tissue parasitism. Am J Pathol.

[6] Viana CEM, Matos DM, Oliveira MF, da Costa AC, Filho TPA, Filho PAM (2021). Immunosuppressive CD14+/HLA-DRlow/‒ monocytes in patients with
Chagas Disease. Acta Trop.

[7] Condamine T, Gabrilovich DI (2011). Molecular mechanisms regulating myeloid-derived suppressor cell
differentiation and function. Trends Immunol.

[8] Dutra WO, Martins-Filho OA, Cançado JR, Pinto-Dias JC, Brener Z, Freeman GL (1994). Activated T and B lymphocytes in peripheral blood of patients
with Chagas’ disease. Int Immunol.

[9] Pérez AR, Morrot A, Berbert LR, Terra-Granado E, Savino W (2012). Extrathymic CD4+CD8+ lymphocytes in Chagas disease: possible
relationship with an immunoendocrine imbalance. Ann N Y Acad Sci.

[10] Martin D, Tarleton R (2004). Generation, specificity, and function of CD8+ T cells in
Trypanosoma cruzi infection. Immunol Rev.

[11] Higuchi Mde L, Gutierrez PS, Aiello VD, Palomino S, Bocchi E, Kalil J (1993). Immunohistochemical characterization of infiltrating cells in
human chronic chagasic myocarditis: comparison with myocardial rejection
process. Virchows Arch A Pathol Anat Histopathol.

[12] Reis DD, Jones EM, Tostes S, Lopes ER, Gazzinelli G, Colley DG, McCurley TL (1993). Characterization of inflammatory infiltrates in chronic chagasic
myocardial lesions: presence of tumor necrosis factor-alpha+ cells and
dominance of granzyme A+, CD8+ lymphocytes. Am J Trop Med Hyg.

